# Characterization of Non-selected Intermolecular Gene Conversion in the Polyploid Haloarchaeon *Haloferax volcanii*

**DOI:** 10.3389/fmicb.2021.680854

**Published:** 2021-06-10

**Authors:** Daniel Wasser, Andreas Borst, Mathias Hammelmann, Katharina Ludt, Jörg Soppa

**Affiliations:** Institute for Molecular Biosciences, Biocentre, Goethe-University, Frankfurt, Germany

**Keywords:** archaea, *Haloferax volcanii*, gene conversion, polyploidy, heterozygous cells, protoplast fusion, unselected segregation

## Abstract

Gene conversion is defined as the non-reciprocal transfer of genetic information from one site to a homologous, but not identical site of the genome. In prokaryotes, gene conversion can increase the variance of sequences, like in antigenic variation, but can also lead to a homogenization of sequences, like in the concerted evolution of multigene families. In contrast to these intramolecular mechanisms, the intermolecular gene conversion in polyploid prokaryotes, which leads to the equalization of the multiple genome copies, has hardly been studied. We have previously shown the intermolecular gene conversion in halophilic and methanogenic archaea is so efficient that it can be studied without selecting for conversion events. Here, we have established an approach to characterize unselected intermolecular gene conversion in *Haloferax volcanii* making use of two genes that encode enzymes involved in carotenoid biosynthesis. Heterozygous strains were generated by protoplast fusion, and gene conversion was quantified by phenotype analysis or/and PCR. It was verified that unselected gene conversion is extremely efficient and it was shown that gene conversion tracts are much longer than in antigenic variation or concerted evolution in bacteria. Two sites were nearly always co-converted when they were 600 bp apart, and more than 30% co-conversion even occurred when two sites were 5 kbp apart. The gene conversion frequency was independent from the extent of genome differences, and even a one nucleotide difference triggered conversion.

## Introduction

Gene conversion is defined as the non-reciprocal transfer of information between two homologous, but not identical DNA sequences. It occurs in all three domains of life, archaea, bacteria, and eukaryotes. However, it has mostly been studied in eukaryotes. A meta-study on gene conversion literature revealed that only 137 out of more than 2,000 studies were performed with bacteria, and only one single study with archaea (Lawson et al., 2009). Gene conversion in eukaryotes has been reviewed repeatedly and will not be discussed here (Tang and Martin, 2007; Duret and Galtier, 2009; McCulloch et al., 2015; Korunes and Noor, 2017). It is a default event in meiosis and has a probability of 10^–6^ –10^–4^ per site in various eukaryotes (Korunes and Noor, 2017). Of course, prokaryotes do not exhibit meiosis, and, therefore, gene conversion must have other functions in prokaryotes than enhancing genetic diversity during meiosis.

**FIGURE 1:**
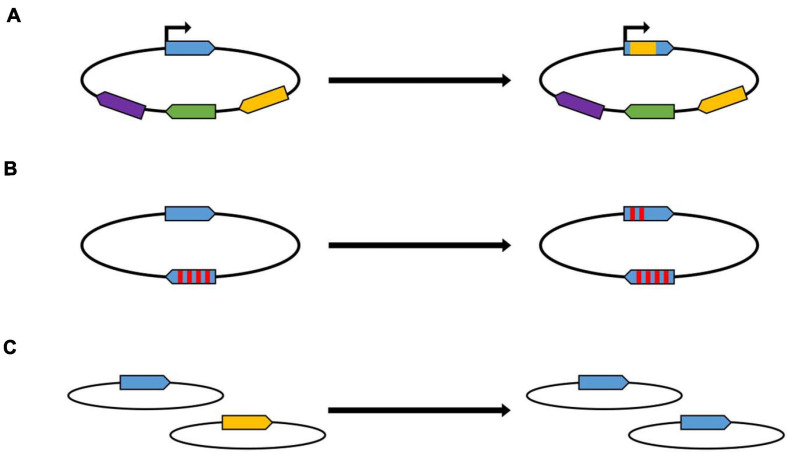
Schematic overview of gene conversion within different biological processes. **(A)** Antigenic variation. The promoter of the allele in the expression site is indicated with an arrow. The different colors indicate homologous, but not identical alleles. **(B)** Concerted evolution of gene families. Two members of a gene family are shown schematically, the red stripes indicate single nucleotide differences between the two genes. **(C)** Equalization of genomes in polyploid prokaryotes. Two genomes are shown schematically, which contain at identical sites two slightly different versions of the same gene.

In many pathogenic bacteria, gene conversion is a mechanism to enhance diversity in a process that is called antigenic variation (Santoyo and Romero, 2005; Vink et al., 2012; Foley, 2015). Antigenic variation has evolved independently in phylogenetically different groups of bacteria. Several molecular mechanisms have been shown to operate, among them gene conversion. Antigenic variation enables pathogenic species to evade the immune response of the host by repeatedly changing surface structures. When the molecular mechanism is gene conversion, the genome typically contains one functional copy of the respective gene, which is preceded by a promoter and is called “expression cassette.” In addition, the genome contains several to many non-expressed copies, which act as donor sites in gene conversion and can overwrite the sequence of the expression site copy (Figure 1A). This changes the sequence of the expressed surface structure and makes the immune response obsolete. The number of silent copies can be very different even in related species, e.g., *Anaplasma marginale* contains 5–7 silent copies, while *A. phagocytophilum* contains about 100 silent copies (Foley, 2015). Notably, the number of possible variants is much higher than the number of silent copies, because typically not the whole ORF is converted, but only small parts, leading to chimeric sequences at the expression site. In these examples, gene conversion results in the generation of variability. The eukaryotic pathogen *Trypanosoma brucei* has taken this principle to the extreme, its genome contains more than 1,500 silent copies of the gene for the variant surface glycoprotein (Vink et al., 2012).

However, gene conversion can also have the opposite effect, i.e., it can lead to the equalization of sequences. This has been observed in a phenomenon called concerted evolution of gene families, which also occurs in various phylogenetically unrelated bacteria (Liao, 2000; Santoyo and Romero, 2005; Espejo and Plaza, 2018). “Concerted evolution” means that the sequences of two or more copies of a gene within one genome are more similar to one another than expected based on the evolutionary history of the gene. One example is the rRNA operon (Espejo and Plaza, 2018). Most bacterial species contain more than one rRNA operon, because during fast growth a high number of ribosomes have to be rapidly synthesized, and, in contrast to protein-coding genes, no translation is involved, and, thus, one step of multiplication in gene expression is missing. For example, the *Escherichia coli* genome contains seven rRNA operons, but the number can be as high as 15 in other bacterial species. Concerted evolution has also been observed in copies of protein-coding genes, well-studied examples include the *tuf* genes in *Salmonella* of the *nifH* genes in *Rhizobium* (Yáñez-Cuna et al., 2016; Paulsson et al., 2017). Also in these cases, not the entire gene is converted, but small patches of several hundred nucleotides (Figure 1B). The conversion frequencies are usually rather low, with rates about 10^–4^ –10^–7^ per site per generation, so that gene conversion experiments include selection steps for conversion events. Gene conversion in concerted evolution of gene families enhances the similarity of the various copies. It can also result in spreading an advantageous mutation, that occurred in one copy, throughout the entire gene family, thereby enhancing the fitness of the organism (Santoyo and Romero, 2005).

During both processes, antigenic variation as well as concerted evolution, gene conversion occurs between different sites on one chromosome, and, thus, it is an intramolecular event. Another process is intermolecular gene conversion between different copies of the chromosome in polyploid prokaryotes. This intermolecular gene conversion leads to the equalization of genome copies with sequence differences, e.g., mutations (Figure 1C). It has hardly been studied, probably because for many years prokaryotes were thought to be monoploid. To our knowledge only two studies with halophilic and methanogenic archaea exists (Hildenbrand et al., 2011; Lange et al., 2011). For both groups it was shown that intermolecular gene conversion is extremely efficient, so that it can be studied even in the absence of selection. Studies about gene conversion in polyploid bacteria are currently not available. However, it can be assumed to operate also in polyploid bacteria, because (1) genome sequencing projects with polyploid bacterial species have not indicated that they might be heterozygous, (2) heterozygous cells have repeatedly been obtained with different bacterial species, but only due to artificial laboratory selection for both types of genomes, and (3) the process has been observed in chloroplasts, the descendants of cyanobacteria (Khakhlova and Bock, 2006).

The present study aimed at a further characterization of non-selected intermolecular gene conversion in the halophilic model species *Haloferax volcanii*. Two genes encoding enzymes involved in carotenoid biosynthesis were chosen for the analysis of gene conversion. Heterozygous *H. volcanii* cells can easily be produced by protoplast fusion of two strains with non-identical genomes. The efficiencies of gene conversion were analyzed using PCR analyses as well as the cell color as a marker for functional carotenoid synthesis. The experiments yielded information about the gene conversion tract length, gene conversion efficiencies between genomes with varying differences, as well as conversion of genes of different species.

## Materials and Methods

### Strains and Culture Conditions

*H. volcanii* was grown in complex medium and in synthetic medium with 0.5% (w/v) glucose as described (Dambeck and Soppa, 2008; Jantzer et al., 2011). The following supplements were added to enable the growth of the respective auxotrophic strains: 50 μg/ml uracil, 50 μg/ml tryptophan, 20 μg/ml thymidine. The *H.* volcanii strains used in this study and characteristic features are listed in Supplementary Table 1.

The *E. coli* strain XL1-blue MRF’ (Agilent Technologies, Waldbronn, Germany) was used for cloning. It was grown in standard SOB complex medium (Hanahan, 1983).

### Generation of Genomic Mutations (Deletions, Insertions, Replacements)

Vectors for the generation of genomic mutations of *H. volcanii* were constructed as described previously (Hammelmann and Soppa, 2008). Supplementary Table 7 gives a list of all primers and characteristic features, e.g., mutated gene. The sequence of all plasmids was verified by sequencing. The mutations were recombined into the genome of *H. volcanii* using the so-called Pop-In-Pop-Out method as described previously (Allers et al., 2004; Jaschinski et al., 2014). *H. volcanii* is polyploid, therefore, it is possible that heterozygous cells arise. Therefore, homozygocity of the mutants was verified by Southern blot analyses and by multicycle PCR with isolated genomic DNA as template.

### Protoplast Fusions

Protoplast fusion was performed essentially as described by Tchelet and Mevarech (1994). In this study, always a tryptophan-auxotrophic strain was fused with a thymidine-auxotrophic strain, which enabled selection for fused cells via growth in the absence of both substances (Ludt, 2018). Exponentially growing *H. volcanii* cells were used to inoculate complex medium with a starting OD_600_ of 0.1. The medium was supplemented with 50 μg/ml tryptophan or 20 μg/ml thymidine for the respective auxotrophic strains. Cultures were grown overnight until they reached an OD_600_ between 1.0 and 1.3. From each of the two cultures 8 × 10^8^ cells were collected by centrifugation (2 min, 8,000 rpm, room temperature) and resuspended in 500 μl sterile 1 M NaCl solution. The suspensions were incubated for 10 min at room temperature, and microscopic inspection was used to verify that they had formed protoplasts. Both suspensions were mixed, centrifuged (10 min, 13,000 rpm, room temperature), and the pellets were carefully resuspended in 168 μl SMT solution (3.5 M NaCl, 150 mM MgCl_2_, 50 mM Tris/HCl, pH 7.2). 132 μl PEG_600_ was pipetted into the lid of the Eppendorf tubes, the tubes were closed and the cell suspensions and the PEG solutions were rapidly mixed by shaking. The suspensions were incubated for 10 min at room temperature, and the (fused) protoplasts were collected by centrifugation (10 min, 6,500 rpm, room temperature). The pellet was resuspended in 100 μl basal salts with 15% (w/v) sucrose. In the first set of experiments, the (fused) protoplasts were directly plated on agar plates with synthetic medium with glucose and uracil. The absence of tryptophan and thymidine selected against both parent strains and allowed solely the growth of fused protoplasts. However, this resulted in a rather high fraction of colonies with red and white sections, indicating that gene conversion had occurred after plating, during the growth of the colonies. Therefore, in all subsequent experiments the (fused) protoplasts were incubated in liquid synthetic medium for 1 day before plating. This resulted in a high fraction of uniform colonies. After plating, the plates were incubated for 5 days at 42^*o*^C to allow colony growth, and for further 3 days at room temperature to enable a better discrimination between red and white colonies.

### Characterization of Unselected Intermolecular Gene Conversion

Most experiments were performed in three biological replicates, and average values and their standard deviations were calculated. Exceptions are the first experiments that were used to optimize the experimental design. When the color of colonies was indicative for the occurrence of gene conversion, the agar plates were incubated as described above, and the numbers of white and red colonies were counted. Partially colored colonies were not counted, therefore, the fractions of red and white colonies did not add up to 100% of all counted colonies (e.g., Supplementary Tables 5, 6). This approach enabled the analysis of thousands of colonies and gave an excellent statistical support.

In addition to color counting and in all other experimental setups analytical PCR analyses were used for the characterization of the genomic organization at the sites of genome differences and putative gene conversion. Equal mixtures of genomes of the two strains used for protoplast fusions were used as internal controls to guarantee that PCR products indicative for both genomic states were faithfully amplified (an example is shown in Figure 3B). In most experiments full gene conversion was observed in (nearly) all analyzed clones. Exceptions were the two experiments with genes *HVO_2524* and *HVO_2528*, which were performed prior to the optimization of the procedure by including a 1 day incubation step before plating (see above). In these cases, band intensities of the PCR products were densitometrically quantified, and clones were counted as gene conversion positive when the intensity of one of the two bands was at least 75%, which indicated that gene conversion had highly progressed (>75%) or had been fully completed (100%). This refers to the results shown in Supplementary Tables 3, 4, in all other experiments, only full conversion was counted.

**FIGURE 2:**
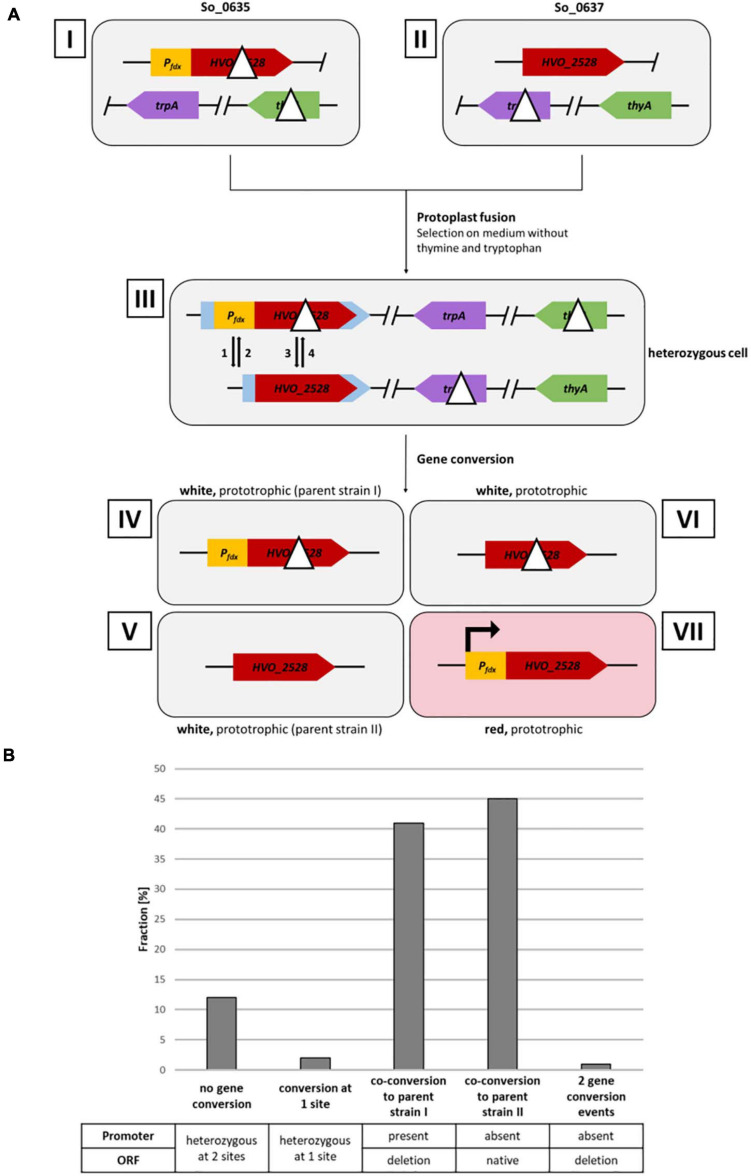
Analysis of gene conversion at two sites with a distance of 626 bp. **(A)** Schematic overview of a gene conversion experiment. Roman numbers indicate the involved input and output strains. Strains I and II are homozygous parent strains, which are fused by protoplast fusion to generate the heterozygous strain III. Strains IV–VII indicate the possible output strains in the absence and presence, respectively, of gene conversion. The Arabic numbers 1–4 indicate possible gene conversion events at the two relevant genomic sites. Gene conversion at the two selection genes *thyA* and *trpA* have been omitted. **(B)** Results of the gene conversion experiment. 130 white clones were analyzed by PCR with primers specific for the two sites of possible gene conversion. The fractions (%) of clones are shown that experienced gene conversion at none, one or two of the sites. One biological replicate was performed.

**FIGURE 3:**
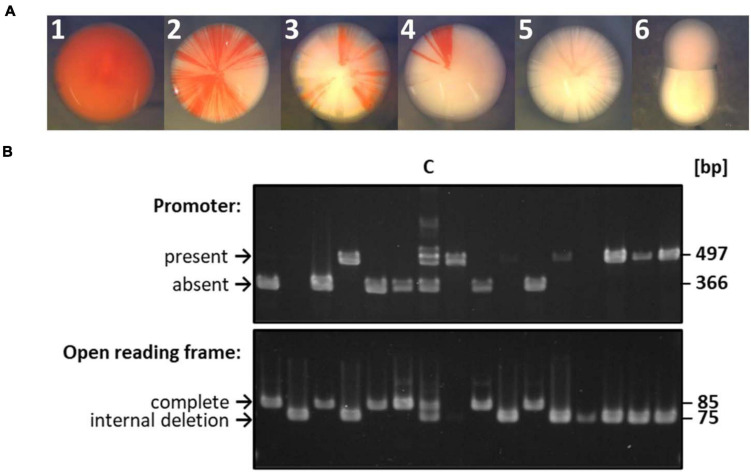
Selected examples of the gene conversion experiment shown in Figure 2. **(A)** Selected examples of colonies observed after a gene conversion experiment. The experiment had been performed as schematically shown in Figure 2. A very early gene conversion event led to colony A, gene conversion events at different times during colony growth led to colonies B to D, and the phenotype of white colonies E and F are not informative about gene conversion. **(B)** Results of PCR analyses of randomly chosen white colonies. Lane C denotes a control that consisted of an equal mixture of the respective longer version (with promoter, no deletion) and shorter version (without promoter, with ORF-internal deletion). The sizes indicative for the longer version and shorter version are indicated.

### Databases and Programs

The genome database Halolex was used to work with the genome sequence of *H. volcanii* and retrieve gene sequences (Pfeiffer et al., 2008). The Clone Manager Professional Suite version 8 (Sci Ed Software, Westminster, Colorado, United States) was used for the planning of experiments, e.g., the design of genome variants. Densitometric quantifications were performed using the freeware ImageJ^1^.

## Results

### Characterization of Unselected Gene Conversion Using a Carotenoid Biosynthesis Gene

In a previous study, we had used the *leuB* and *trpA* genes for the characterization of gene conversion in *H. volcanii* (Lange et al., 2011). It was revealed that gene conversion is highly efficient. The encoded enzymes are involved in the biosynthesis of the amino acids leucine and tryptophan. Therefore, in the presence of both amino acids, the presence or absence of the genes should not give a selective advantage or disadvantage. Nevertheless, gene conversion was found to be very effective under these conditions. However, there is a very strong selection pressure in the absence of the amino acids, and the situation is not very clear at low amino acid concentrations. Therefore, we wanted to establish another gene system for the further characterization of gene conversion. We had shown that the deletion of the gene *HVO_2528*, which encodes the carotenoid biosynthesis enzyme phytoene dehydrogenase, results in the formation of white cells, while the wildtype forms red cells (Maurer et al., 2018). Growth of the deletion mutant and the wildtype were indistinguishable in several media during incubation in the dark. Therefore, the gene *HVO_2528* was chosen for the further characterization of unselected gene conversion in *H. volcanii.*

Two versions of the gene were generated: (1) one version with a strong constitutive promoter and an internal deletion in the open reading frame (ORF), which resulted in the production of a non-functional protein, and (2) one version with an intact ORF, which was not preceded by a promoter and, thus, was not expressed. In different strains, the two versions were introduced into the chromosome of *H. volcanii* at the *HVO_1752* locus, which has no obvious functional role. A deletion mutant of *HVO_1752* grew indistinguishable from the wildtype under a large number of tested conditions (Dambeck, 2010). In both strains the gene *HVO_2528* had been deleted at its native position. Each of the two strains contained an additional deletion, the *thyA* gene encoding an enzyme for thymine biosynthesis in one strain, and the *trpA* gene encoding an enzyme for tryptophan biosynthesis in the other strain. Supplementary Figure 1 gives an overview of the genomic organization of the wildtype and the two derived strains at the relevant genomic sites.

Figure 2A gives a schematic overview of a gene conversion experiment. The protoplast fusion technique (Rosenshine and Mevarech, 1989; Tchelet and Mevarech, 1994; Ludt, 2018) was used to fuse the two strains described above. Growth in synthetic medium in the absence of thymine and tryptophan selected for fused cells, because neither of the two parent strains can grow in the absence of these substances. The expected possible outcomes of gene conversion events are indicated by the arrows 1 – 4 in the schematic heterozygous cell (cell III in Figure 2A). In half of the cases, cells are expected to be white (events 2 and 3), while in the other half of the cases, the cells become red (events 1 and 4). Figure 3A shows selected examples of colonies that could be observed. These included red colonies, indicating that gene conversion happened very early, before growth of the colony started (Figure 3A1). In addition, partially red colonies were observed, which indicated that gene conversion occurred at different stages during the growth of the colony (Figure 3A2–4). However, the vast majority of colonies were white (Figure 3A5,6). Only 12 of more than 8,500 colonies were red or had red segments. This very small fraction of only 0.14%, which seemed to indicate that gene conversion is extremely rare in *H. volcanii*, strongly contradicting earlier results, which had shown that gene conversion in *H. volcanii* is extremely efficient (Lange et al., 2011). Therefore, 130 white colonies were randomly chosen, and the genomic organization at the two relevant genomic sites were analyzed by PCR. The results for a few selected clones are shown in Figure 3B, and all results are summarized in Figure 2B and Supplementary Table 2. It turned out that the vast majority of 86% had experienced gene conversion and had become homozygous at both sites. The gene conversion tract was so long that in these cases co-conversion had occurred at both sites, despite their distance of 626 bp. This means that when the promoter was transferred to the promoter-less version (event 1), simultaneously the deletion within the ORF was transferred to the native ORF (event 3), and the resulting strain (strain IV in Figure 2A) became identical to one of the parent strains (strain I). Also events 2 and 4 occurred simultaneously, resulting in a strain (strain V) that was identical to the other parent strain (strain II in Figure 2A). The expected gene conversion at only one of the two sites was extremely rare, only 1 of the 135 clones was promoterless and had the deletion version of the ORF, and, as stated above, only 0.14% had a promoter in front of the native ORF and had become red. Only 12% were still heterozygous at both sites and had thus not experienced gene conversion. Taken together, gene conversion was very efficient, but the gene conversion tract was so long that in nearly all cases co-conversion at both sites occurred, preventing the formation of red clones. Therefore, the next aim was to quantify the co-conversion frequency at two genomic sites that were separated by a much larger distance.

### Quantification of the Co-conversion Frequency at Two Different Genes

**FIGURE 4:**
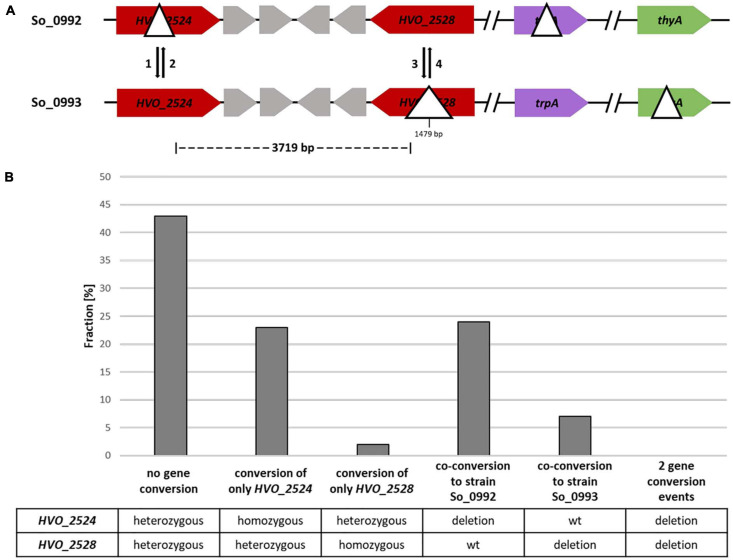
A gene conversion experiment including two sites with a distance of 3,719 bp. **(A)** Design of the experiment. The sizes of the internal deletions and the genomic distance between them are indicated. The numbers 1–4 indicate the possible gene conversion events. **(B)** Results of the experiment. Ninety five randomly chosen white clones were analyzed by PCR with primers that were specific for the two possible gene conversion sites. The fractions (%) of clones are shown that exhibited gene conversion at none, one or two of the sites. One biological replicate was performed.

For the analysis of the co-conversion frequency at two more distant sites two genes were chosen, both of which encode enzymes involved in carotenoid biosynthesis: (1) gene *HVO_2528* that had been already used for the experiment described above, and (2) gene *HVO_2524* that encodes phytoene synthase. Two strains were generated that contained a large ORF-internal deletion in one or the other of the two genes (Figure 4A). The two genes are separated by four other genes, and the distance of the two potential gene conversion sites is 3.7 kbp. In addition, the strains carried a deletion in either the *trpA* gene or the *thyA* gene, and, thus, they could be used for gene conversion experiments that include protoplast fusion and selection of fused, heterozygous clones. As in the experiment described above, also in this experimental design red colonies can only be generated when gene conversion occurs at only one of the two sites. The fraction of (at least partially) red colonies was about 6.6% (40 of 607 colonies), and, thus, a single site conversion event has a much higher frequency when the distance of two sites is 3,700 bp than when it is 626 bp. Ninety five white clones were chosen randomly, and the organization at the two sites was analyzed by PCR. The results are summarized in Figure 4B and Supplementary Table 3. Single site conversion in the direction of the ORF-internal deletion variant had occurred in 25%.

In 31% of all clones co-conversion at both sites had occurred, showing that a considerable fraction of gene conversion tracts in *H. volcanii* are longer than 4 kbp (Figure 4B and Supplementary Table 3). This is much longer than the typical gene conversion tract lengths in bacteria (Yáñez-Cuna et al., 2016; Paulsson et al., 2017). The fraction of clones without gene conversion, which were still heterozygous at both sites, was about 43%.

**FIGURE 5:**
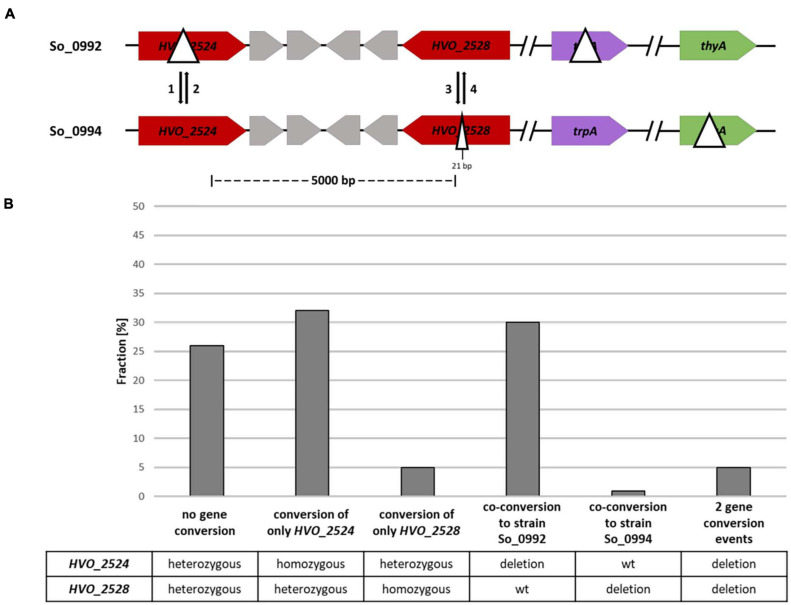
A gene conversion experiment including two different sites with a distance of 5,000 bp. **(A)** Design of the experiment. The sizes of the internal deletions and the genomic distance between them are indicated. The numbers 1–4 indicate the possible gene conversion events. **(B)** Results of the experiment. Ninety three randomly chosen white clones were analyzed by PCR with primers that were specific for the two possible gene conversion sites. The fractions (%) of clones are shown that exhibited gene conversion at none, one or two of the sites. One biological replicate was performed.

For a further gene conversion experiment an additional strain was generated that contained a very small deletion of 21 bp in the ORF of the *HVO_2528* gene instead of the 1479 bp deletion used in the experiment described above. The two strains used for the gene conversion experiment are schematically shown in Figure 5A. In this case the distance between the two possible gene conversion sites was 5 kbp. The fraction of (at least partially) red colonies was 9.4% (72 out of 762 clones). 93 white clones were randomly chosen and their genomic organization at the two sites was analyzed by PCR (Figure 5B and Supplementary Table 4). Conversion at only one of the two sites was observed in 37% of all cases, an even higher fraction than the 25% observed in the experiment described above. Therefore, this result underscores that the frequency of conversion of only one site is much higher when the two sites have a larger distance. Unexpectedly, the conversion frequency solely at *HVO_2528* was a bit higher when the deletion was only 21 bp then when it was much larger with 1,479 bp (5% vs. 2%). However, the numbers were very small, did not reach statistical significance, and it seemed desirable to clarify this question using an experimental design that enables the analysis of much higher number of clones and that is not complicated by a second gene conversion site (see below).

Co-conversion at both sites had occurred in 31% of all clones, a value identical to the 31% observed in the first experiment with the two long-distance sites. In very few cases, two gene conversion events in opposite directions were observed, leading to a strain with homozygous deletions at both sites. In 26% of all cases both sites were still heterozygous, and thus, no gene conversion had occurred (at least no full conversion to homozygocity). The fractions of clones without obvious gene conversion differed in the three experiments (12, 43, 26%), and thus it seemed that a further optimization of the experimental design seemed necessary.

### The Extent of Genome Differences and Gene Conversion Efficiencies

**FIGURE 6:**
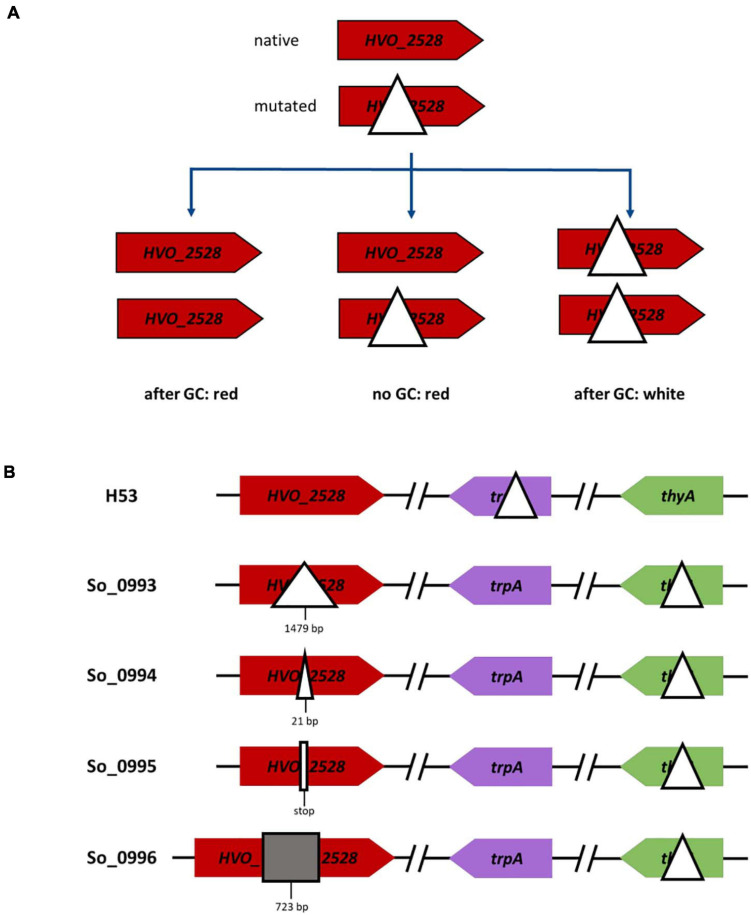
Schematic overview of gene conversions experiments involving the wildtype genome and genomes with various modifications at one site. **(A)** The versions of gene *HVO_2528* in the two parent strains are shown on top, the three possible output strains are shown below. **(B)** Overview of one parent strain (on top) and the four versions of the second parent strain (below), which contained different mutations in gene *HVO_2528*.

Next, we wanted to clarify whether the extent of the genome difference in heterozygous cells influences the conversion frequency. In addition, the differences should be restricted to only one conversion site. The new experimental design is schematically shown in Figure 6A. A strain with a wildtype copy of the gene *HVO_2528* was fused with a strain with an ORF-internal deletion or another mutation, resulting in a heterozygous cell that was capable of carotenoid synthesis. Further cultivation of the heterozygous cells could have three possible outcomes, i.e., gene conversion in the direction of the native ORF as well as the absence of gene conversion resulted in red cells, while gene conversion in the direction of the deletion resulted in white cells. The fraction of white cells can easily be determined in large populations, and thus this assay was ideally suited for the exact quantification of conversion frequencies.

**FIGURE 7:**
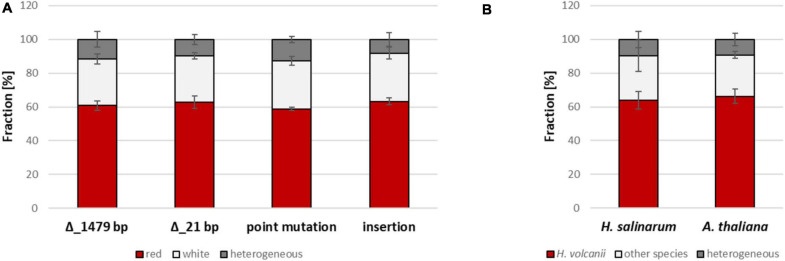
Results of gene conversion experiments between the wildtype genome and genomes with various modifications at one site. **(A)** Results of the gene conversion experiments that are schematically shown in Figure 6 are summarized. Between 2,617 and 6,324 colonies were analyzed (Supplementary Table 5), and the fractions of white colonies, red colonies, and heterogenous colonies were quantified. The average fractions (%) of four biological replicates and their standard deviations are shown. A statistical analysis of significance was performed (one-way ANOVA) and the differences between the fractions of red and white clones were not significant (*P* > 0.05). **(B)** The results of two experiments are shown in which instead of mutated version of *HVO_2528* orthologous genes from other species were used, as indicated. For the *H. salinarum* ortholog, the 222 resulting clones were analyzed by PCR, for the *A. thaliana* ortholog, the colors of 5,491 colonies were analyzed. The average fractions (%) of, respectively, three biological replicates (*H. salinarum*) and four biological replicates (*A. thaliana*) and their standard deviations are shown. A statistical analysis of significance was performed (unpaired *t*-test) and the differences were not significant in both cases (*P* > 0.05).

Four different mutated versions of *HVO_2528* were compared, which contained, respectively, ORF-internal deletions of 1,479 bp and 21 bp, a point mutation that resulted in a stop codon, and an insertion of 723 bp (Figure 6B). The results are summarized in Figure 7A and Supplementary Table 5. Surprisingly, there were no significant differences between the fractions of white cells, in fact, they were nearly identical. It was remarkable that even a single nucleotide difference in a chromosome of 2.85 Mbp could trigger gene conversion in *H. volcanii*, and, even more, that the efficiency was the same as a 1,479 bp deletion. PCR analyses of about 100 randomly chosen red clones revealed that nearly all of them were homozygous, with very few exceptions. These results again underscored that gene conversion in *H. volcanii* is extremely efficient. However, it remains unclear what determines the direction of gene conversion, because ORF-internal deletions and the ORF-internal insertion resulted in the same fraction of white cells.

### Gene Conversion Between Genes From Different Species

In a last experiment, gene conversion between homologous genes from different species was analyzed. First, the gene for the phytoene dehydrogenase from *Halobacterium salinarum* was chosen (*OE3381R*), which is 74% identical to the *HVO_2528* gene of *H. volcanii*. A strain was generated that contained the *OE3381R* gene instead of the *HVO_2528* in its genome. The resulting cells were red, showing that the *Halobacterium* enzyme was fully functional in the *Haloferax* cytoplasm, despite the lower intracellular salt concentration of the latter species (2.1 M vs. 4 M NaCl). Two strains with genes from *H. volcanii* and *H. salinarum*, respectively, were fused to analyze the occurrence of gene conversion. Of course in this case the color could not be used to quantify the occurrence and efficiency of gene conversion, therefore, more than 200 randomly chosen clones were analyzed by PCR (Figure 7B and Supplementary Table 6). 90% of the clones had become homozygous, a majority of 64%contained the *H. volcanii* gene, while 26% contained the *H. salinarum* gene. Sequencing of a few examples revealed that gene conversion did not result in the formation of chimeric genes, but that the whole ORF had been converted, again underscoring that the gene conversion tracts in *H. volcanii* are very long. Based on this result, this can also be expected for the alternative event, i.e., homologous recombination involving reciprocal crossover.

Next, a phytoene dehydrogenase gene from a very distantly related species was chosen, i.e., the gene *AT4G14210.1* from the plant *Arabidopsis thaliana*. The gene has a sequence identity of only 43% to the *H. volcanii* gene, while the average identity of any two totally unrelated sequences is 25%. A strain was generated that contained the *A. thaliana* gene instead of *HVO_2528* in its chromosome. The strain was white, showing that the plant enzyme could not replace the haloarchaeal enzyme, most probably because proteins from mesohalic species do not fold properly in the high salt cytoplasm of haloarchaea. The strain was fused with a strain containing the native *HVO_2528* gene, and the color of nearly 5,500 clones was determined (Figure 7B and Supplementary Table 6). 24.5% of the clones were white, showing that the plant gene had converted the haloarchaeal gene and the clones were homozygous. Sequencing of a few randomly chosen red and white clones revealed that in both directions the whole ORF had been converted, and chimeric genes could not be detected. Taken together, these results show that gene conversion in *H. volcanii* can occur between genes of related, but also very distant species, and that the gene conversion tracts are very long.

## Discussion

Intermolecular gene conversion in polyploid prokaryotes has hardly been studied, only two studies have been published with halophilic and methanogenic bacteria and one with chloroplasts (Khakhlova and Bock, 2006; Hildenbrand et al., 2011; Lange et al., 2011). In the present study we established the use of genes encoding enzymes involved in carotenoid biosynthesis for the characterization of unselected intermolecular gene conversion in the polyploid haloarchaeon *H. volcanii*. The species is ideally suited for the analysis of gene conversion, because (1) it can be cultivated in various media very easily and has a doubling time around 3 h, (2) the genome can be changed very easily due to an efficient genetic system, and (3) heterozygous cells with different genomes can be generated very easily using a protoplast fusion technique. In a first approach, two white strains were fused with the expectation that gene conversion would lead to the restoration of carotenoid biosynthesis and thus converted cells could be recognized based on their red color. However, it turned out that the two sites of designed genome differences, which had a distance of 626 bp, were nearly always co-converted and only a very small fraction of 0.14% was red. This is in stark contrast to the gene conversion tract lengths of only a few hundred nucleotides that have been determined in concerted evolution of multigene families in bacteria (Santoyo et al., 2005; Castellanos and Romero, 2009; Yáñez-Cuna et al., 2016; Paulsson et al., 2017). For example, only 50% co-conversion has been observed for two sites at a distance of 600 bp at the *tuf* genes of *Salmonella*, and the co-conversion rate dropped to 15% when two sites had a distance of 1,000 bp (Paulsson et al., 2017). The gene conversion tract lengths observed in antigenic variation in bacteria are also very short, and typically only parts of the respective genes are converted (Vink et al., 2012). Similarly, the mean gene conversion tract length in the meiosis of *Drosophila melanogaster* was determined to be 476 bp (Miller et al., 2012). For gene conversion in the meiosis of *Saccharomyces cerevisiae*, a somewhat higher value of 1.5 kbp has been obtained (Chakraborty et al., 2018). The very high co-conversion rate in the experiment described above indicated that the value might well be considerable larger in *H. volcanii* than in all those processes in bacteria and eukaryotes.

For a further characterization of gene conversion tract lengths in *H. volcanii*, two sites with a distance of 3.7– 5 kbp, respectively, were chosen, and the co-conversion rates were found to be higher than 30%. Conversion at only one of the two sites was found in 25 and 37% of all cases, respectively, showing that the tracts ended between the two sites. Of course this does not mean that the conversion tracts in these latter cases were smaller than 3.7 or 5 kbp, because the position of the second end of the conversion tracts was unknown. One interspecies experiment had shown that the tracts of homologous recombination (gene conversion or crossover recombination) in haloarchaea can be extremely long (Naor et al., 2012). Hybrids between the two species *H. volcanii* and *Haloferax mediterranei* had been selected using a double-selection against both species, and their genome content was characterized. A further selection led to the identification of hybrid cells in which recombination between the genomes of the two species had occurred. Very large fragments between 310 and 530 kbp (average 475 kbp) had been exchanged. However, the fraction of interspecies hybrid formation was very small (10^–4^ –10^–5^), and only 10% of all hybrids showed recombination, and, therefore, this result might not be transferrable to the mechanism of unselected intermolecular gene conversion described above. In future experiments co-conversion rates between more sites of variable distance will be quantified to determine the typical tract lengths in this process in *H. volcanii*.

In a bioinformatics approach, genome sequences of 21 haloarchaeal species were used to predict probable horizontal gene transfer (HGT) events during the evolution of haloarchaea (Williams et al., 2012). Only HGT within the haloarchaea were analyzed. In total, 1,610 HGT events could be detected. 98% of the events were restricted to the transfer of a single gene (1,751 events), and the simultaneous transfer of two or more genes was extremely rare. These results do not seem to be in agreement with the results of the interspecies mating experiment described above (Naor et al., 2012) or with the results of this study, which shows that gene conversion tracts are typically long. Future experimental and bioinformatics analyses should aim to resolve this apparent discrepancy.

A further big difference between concerted evolution of gene families in bacteria and intermolecular gene conversion in *H. volcanii* is the efficiency of the respective process. For concerted evolution rates of 10^–3^ –10^–8^ have been reported, while, in stark contrast, between 60 and 90% of all colonies showed gene conversion in the experiments performed in this study (Figures 2, 4, 5, 7 and Supplementary Tables 2–6). The difference might be due to species-specific rates in homologous recombination. A bioinformatic meta-analysis of the results of Multi-Locus-Sequence-Typing (MLST) studies indicated that the rates of homologous recombination in different prokaryotic species differ by more than three orders of magnitude (Vos and Didelot, 2009). 46 bacterial and 2 archaeal species were analyzed, and the two archaeal species had intermediate rates, indicating that a domain-specific difference between bacteria and archaea does not exist. It is tempting to speculate that the large difference is due to the different biological roles of the two processes. On the one hand, sequences of two or more alleles are equalized in monoploid bacteria, and the process does not seem to need a very high efficiency per generation. On the other hand, multiple copies of whole genomes are equalized in a polyploid species, and, in this case, a high efficiency seems to be of advantage.

In fact a theory named “Muller’s ratchet” has predicted that—for theoretical reasons—it is totally impossible that asexual polyploid species exist (Muller, 1964; Carlson, 2013). The reasoning was that in the various genome copies in polyploid species unavoidably deleterious mutations in essential genes must accumulate up to a point that the rate of non-viable daughter cells increase and the species goes extinct. Of course, a highly efficient correction of mutations via intermolecular gene conversion would be an escape from Muller’s ratchet, which has been discussed occasionally (Lehman, 2003; Soppa, 2013; Maciver, 2016). If that would be true, it is tempting to speculate that highly efficient intermolecular gene conversion might not only be found in haloarchaea, but in many polyploid species of archaea and bacteria. It has been proposed that this mechanism also operates in eukaryotes, namely in polyploid amoebae (Maciver, 2016).

On first sight it was unexpected that the extent of genome difference had no influence on the efficiency of gene conversion, and that also a single nucleotide difference could trigger gene conversion. However, also point mutations can lead to a loss of function of a gene product and can be deleterious. Therefore, a mechanism that detects and acts on any differences of genome copies might have evolutionary advantages compared with a mechanism that detects only large differences. It will be interesting to compare the conversion efficiencies of various types of genome differences in other species of polyploid prokaryotes.

The experiments of this study were designed in the expectation that differences in genomes initiate gene conversion in *H. volcanii*. It should be noted that an alternative explanation exists, which would also be compatible with the observed independence of the size of the differences. It might be that *H. volcanii* chromosomes experience a high fraction of double strand breaks (DSBs). If DSB repair occurs via homologous recombination, the repair leads to gene conversion from the genome molecule that was used as repair template to the molecule that had experienced that DSB. As long as the involved genome molecules have identical sequence, this “gene conversion” remains unnoticed. In this view the observed gene conversion would just be as side effect of the very efficient DSB repair, and there is no need to detect sequence differences. In future experiments it will be tested whether the experimental induction of DSBs has an influence on the efficiency of gene conversion.

The efficiencies of gene conversion were overall high, but were not identical at different sites and in different directions. For example, the conversion rate at only HVO_2524 was much higher than at only HVO_2528 (Figures 4, 5 and Supplementary Tables 3, 4), and the conversion of genes of different species was about 65% in the direction of the native *H. volcanii* gene (Figure 7B and Supplementary Table 6). The reasons are not clear and need further investigation. It has been proposed that gene conversion is GC-biased in eukaryotes as well as in most if not all bacteria (Pessia et al., 2012; Lassalle et al., 2015). This would be compatible with the high GC content of haloarchaeal genomes. On the other hand, also the opposite has been reported, i.e., the gene conversion is AT-biased in bacteria (Bobay and Ochman, 2017). The experiment with genes of different species indicates that the GC-content might not be a crucial factor for the direction of gene conversion in *H. volcanii*. Genes of species with extremely different GC contents were used, *H. salinarum* has a GC content of 68%, very similar to that of *H. volcanii* (65%), while *A. thaliana* has a much lower GC content of only 36%. Also the GC contents of the respective genes were very different with 66.1% (*H. volcanii*), 68.8% (*H. salinarum*), and 43.7% (*A. thaliana*). Nevertheless, in both cases the direction of gene conversion in favor of the native *H. volcanii* copy were very similar (63.9% vs. 66.3%). Cleary, it remains to be unraveled what DNA features or motifs determine the efficiency and direction of gene conversion in *H. volcanii.*

## Conclusion

An experimental approach to characterize unselected intermolecular gene conversion in *H. volcanii* has been established, which makes use of two genes that encode enzymes involved in carotenoid biosynthesis. The efficiencies of gene conversion were found to be extremely high, which leads to an effective equalization of the many genome copies in this polyploid species. Gene conversion tracts in *H. volcanii* are much longer than in concerted evolution or antigenic variation in bacteria. Even single nucleotide differences are capable of triggering gene conversion, and the effect is independent from the extent of genome difference. Gene conversion occurs also between homologous genes of very different species. The experimental approach is an excellent basis for further studies, e.g., aiming at the identification of involved proteins and at the unraveling of the molecular mechanism. As efficient intermolecular gene conversion counteracts Muller’s ratchet, it is proposed that it acts not only in haloarchaea, but also in many additional polyploid archaeal and bacterial species.

## Data Availability Statement

The original contributions presented in the study are included in the article/Supplementary Material, further inquiries can be directed to the corresponding author/s.

## Author Contributions

DW, KL, and JS designed the study. DW, MH, and KL performed the experiments. DW, AB, KL, and JS analyzed the data. JS wrote a draft version of the manuscript. All authors contributed to and agreed with the manuscript.

## Conflict of Interest

The authors declare that the research was conducted in the absence of any commercial or financial relationships that could be construed as a potential conflict of interest.

## Footnotes

1 https://imagej.nih.gov/ij/

## Supplementary Material

Supplementary Figure 1 Schematic overview of the relevant genomic regions of strains involved in the first gene conversion experiment.

Supplementary Table 1 Overview of strains and their genotypes.

Supplementary Table 2 Results of the gene conversion experiment shown in Figure 2.

Supplementary Table 3 Results of the gene conversion experiment shown in Figure 4.

Supplementary Table 4 Results of the gene conversion experiment shown in Figure 5.

Supplementary Table 5 Results of the gene conversion experiment shown in Figures 6, 7A.

Supplementary Table 6 Results of the gene conversion experiment shown in Figure 7B.

Supplementary Table 7 List of oligonucleotides used for mutant generation, probe construction, and sequencing.
