# Supplementary material for: Characterization of Non-selected Intermolecular Gene Conversion in the Polyploid Haloarchaeon Haloferax volcanii
Source: Front Microbiol. 2021 Jun 10;12:680854. doi: 10.3389/fmicb.2021.680854 (PMC8223754; doi:10.3389/fmicb.2021.680854)
Supplement: Supplementary Figure 1 — Schematic overview of the relevant genomic regions of strains involved in the first gene conversion experiment. [file Table_1.docx]

Supplementary Material


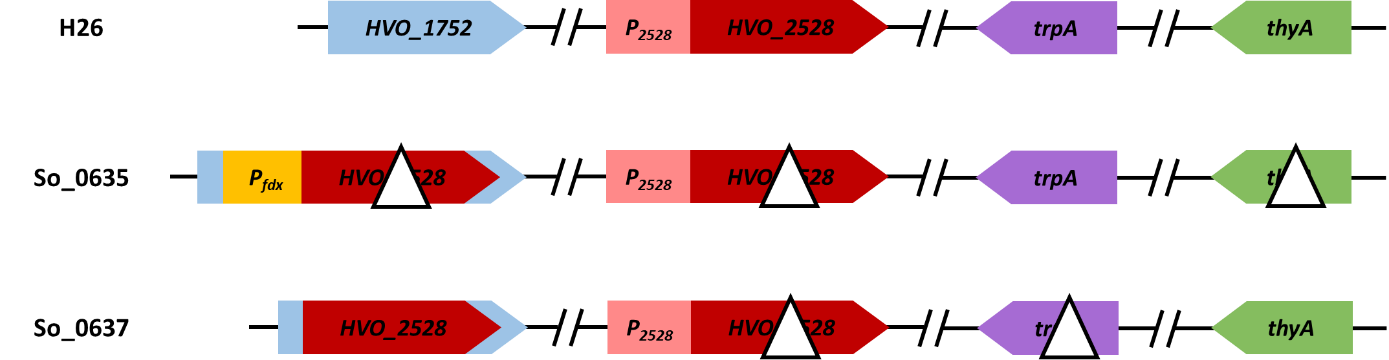


**Supplementary Figure 1: Schematic overview of the relevant genes of the involved strains of the first experiment.** The wildtype is shown on top, two strains applied for gene conversion experiments are shown below. The genes are designated by gene names (*trpA, thyA*) or by the HVO-numbers. Internal deletions are indicated by white triangles.

**Supplementary Table 1: Overview of strains and their genotype.** The relevant genes for the analysis of unselected gene conversion are shown in bold.

| **Strain** | **Genotype** | **Source** |
| --- | --- | --- |
| H26 | *ΔpyrE2* | (Allers *et al,* 2004) |
| H53 | *ΔpyrE2; ΔtrpA* | (Allers *et al,* 2004) |
| So_0635 | *ΔpyrE2; ΔthyA; ΔcrtD;* ***HVO_1752::P_fdx_-HVO_2528mut*** | (Ludt, 2018) |
| So_0637 | *ΔpyrE2; ΔtrpA; ΔcrtD;* ***HVO_1752::HVO_2528*** | (Ludt, 2018) |
| So_0992 | *ΔpyrE2; ΔtrpA;* ***ΔHVO_2524_990nt*** | This study |
| So_0993 | *ΔpyrE2; ΔthyA;* ***ΔHVO_2528_1479nt*** | This study |
| So_0994 | *ΔpyrE2; ΔthyA;* ***ΔHVO_2528_21nt*** | This study |
| So_0995 | *ΔpyrE2; ΔthyA;* ***HVO_2528_stop*** | This study |
| So_0996 | *ΔpyrE2; ΔthyA;* ***iHVO_2528_723nt*** | This study |
| So_0997 | *ΔpyrE2; ΔthyA;* ***HVO_2528::OE3381R (sal)*** | This study |
| So_0998 | *ΔpyrE2; ΔthyA;* ***HVO_2528::AT4G14210.1 (ara)*** | This study |

**Supplementary Table 2:** **Genomic organization at two possible gene conversion sites in 130 randomly chosen white clones.** A schematic overview of the experiment is shown in Figure 2A. The analysis was performed via PCR with primers specific for the two sites, respectively. Selected PCR results are shown in Figure 3B (n.a.: not applicable).

| Promoter | ORF | No. | Fraction | Remark |
| --- | --- | --- | --- | --- |
| heterozygous at 2 sites | | 16 | 12 % | no gene conversion |
| heterozygous at 1 site | | 2 | 2 % | conversion of only one marker |
| present | deletion | 53 | 41 % | co-conversion to parent strain I |
| absent | native | 58 | 45 % | co-conversion to parent strain II |
| absent | deletion | 1 | 1 % | two gene conversion events |
| present | native | n.a. | n.a. | red, not in PCR analysis |

**Supplementary Table 3:** **Genomic organization at two possible gene conversion sites in 95 randomly chosen white clones.** A schematic overview of the experiment is shown in Figure 4A. The genes *HVO_2524* and *HVO_2528* encode enzymes involved in carotenoid biosynthesis. Two large deletions were generated, which have a distance of 3.7 kbp. The analysis was performed via PCR with primers specific for the two sites, respectively (n.a.: not applicable).

| *HVO_2524* | *HVO_2528* | No. | Fraction | Remark |
| --- | --- | --- | --- | --- |
| heterozygous | heterozygous | 41 | 43 % | no gene conversion |
| wt | heterozygous | 0 | 0 % | conversion of only *HVO_2524* |
| deletion | heterozygous | 22 | 23 % | conversion of only *HVO_2524* |
| heterozygous | wt | 0 | 0 % | conversion of only *HVO_2528* |
| heterozygous | deletion | 2 | 2 % | conversion of only *HVO_2528* |
| deletion | wt | 23 | 24 % | co-conversion to strain So_0992 |
| wt | deletion | 7 | 7 % | co-conversion to strain So_0993 |
| deletion | deletion | 0 | 0 % | two gene conversion events |
| wt | wt | n.a. | n.a. | red, not in PCR analysis |

**Supplementary Table 4:** **Genomic organization at two possible gene conversion sites in 93 randomly chosen white clones.** A schematic overview of the experiment is shown in Figure 5A. The genes *HVO_2524* and *HVO_2528* encode enzymes involved in carotenoid biosynthesis. Two deletions of very different sizes were generated, which have a distance of 5 kbp. The analysis was performed via PCR with primers specific for the two sites, respectively (n.a.: not applicable).

| *HVO_2524* | *HVO_2528* | No. | Fraction | Remark |
| --- | --- | --- | --- | --- |
| heterozygous | heterozygous | 24 | 26 % | no gene conversion |
| wt | heterozygous | 0 | 0 % | conversion of only *HVO_2524* |
| deletion | heterozygous | 30 | 32 % | conversion of only *HVO_2524* |
| heterozygous | wt | 1 | 1 % | conversion of only *HVO_2528* |
| heterozygous | deletion | 4 | 4 % | conversion of only *HVO_2528* |
| deletion | wt | 28 | 30 % | co-conversion to strain So_0992 |
| wt | deletion | 1 | 1 % | co-conversion to strain So_0994 |
| deletion | deletion | 5 | 5 % | two gene conversion events |
| wt | wt | n.a. | n.a. | red, not in PCR analysis |

**Supplementary Table 5:** **Fractions of white and red clones after a gene conversion experiment with four strains carrying different mutations in gene *HVO_2528.*** A schematic overview of the experiment is shown in Figure 6.

|  | | **White clones** | | **Red clones** | | **Total** |
| --- | --- | --- | --- | --- | --- | --- |
| **Strain** | **Mutation in *HVO_2528*** | **No.** | **Fraction** | **No.** | **Fraction** | **No.** |
| So_0993 | deletion of 1479 bp | 816 | 28.4 ± 2.8 % | 1761 | 61.3 ± 2.9 % | 2872 |
| So_0994 | deletion of 21 bp | 725 | 27.7 ± 1.9 % | 1625 | 62.1 ± 3.8 % | 2617 |
| So_0995 | point mutation (stop) | 1799 | 28.4 ± 2.6 % | 3725 | 58.9 ± 0.9 % | 6324 |
| So_0996 | insertion of 723 bp | 1772 | 29.6 ± 3.5 % | 3836 | 64.1 ± 2.0 % | 5987 |

**Supplementary Table 6: Characterization of clones after gene conversion with heterologous genes from *H. salinarum* and *A. thaliana*.** The characterization was performed either via PCR (*H. salinarum*) or phenotypically (*A. thaliana*).

|  |  | **Homozygous gene from** | | | |  |
| --- | --- | --- | --- | --- | --- | --- |
|  |  | ***H. volcanii*** | | **second species** | | **Total** |
| **Strain** | **Gene from species** | **No.** | **Fraction** | **No.** | **Fraction** | **No.** |
| So_0997 | *H. salinarum* (*OE3381R*) | 142 | 64 ± 5 % | 58 | 26 ± 9 % | 222 |
| So_0998 | *A. thaliana* (*AT4G14210.1*) | 3755 | 68.4 ± 4.3 % | 1347 | 24.5 ± 2.0 % | 5491 |

**Supplementary Table 7: Overview of oligonucleotides used in this study** (*1: Generation of mutants, *2: Analysis, *3: Sequencing).

| Name | Sequence | Use |
| --- | --- | --- |
| HVO_2528 P1 | ATGCGCCCGTCGTCGGCCATG | 1 |
| HVO_2528 P2 | CTCATCTCGCGTCGACAGAATTCATGTCCGTTCGTAAGGACGTAGAGAAC | 1 |
| HVO_2528 P3 | CATGAATTCTGTCGACGCGAGATGAGCGCCGATATGGC | 1 |
| HVO_2528 P4 | AGACCGAGTCGAGAAGCGGCGTCG | 1 |
| HVO_2918 P1 for | CGGGGAGAGGCGGTTTGCGTATTGGGAGGTGGTCGTCCTTGTG | 1, 2 |
| HVO_2918 P2 rev | CCGGGTCGTAGTCGCGGAGGACGATATATTGGCGCATGAGAGG | 1 |
| HVO_2918 P3 for | CAGGGCACCTCTCATGCGCCAATATATCGTCCTCCGCGACTAC | 1 |
| HVO_2918 P4 rev | CGAACGACCGAGCGCAGCGAGTCAGCCGAGGAACTGCTCGTAG | 1, 2 |
| pKS13 for | ATGAATTCTGTCTCGGCTC | 1 |
| pKS13 rev | TCATCTCGCGTCTTCGG | 1 |
| Mut_2528_for | CCTCACCGACTACATGTGTTTCGGTACTCG | 1 |
| Mut_2528_rev | CGAGTACCGAAACACATGTAGTCGGTGAGG | 1 |
| oadh-F1 | CGATTATCTCTCGGTGATGTGTGGGTTCACACG | 1 |
| oadh-F2 | GGGTTTTATCCACGGCATGCCGTTTTCGTATATAATTTACTTTGACTCGGA | 1 |
| oadh-F3 | AAGTAAATTATATACGAAAACGGCATGCCGTGGATAAAACCCCTCGTTGAC | 1 |
| oadh-F4 | CGAGACAGAATTCATCACTGCAGAGTTCGGCTTCGTTGATTCATC | 1 |
| oadh-F5 | CCGAACTCTGCAGTGATGAATTCTGTCTCGGCTCTCGACTCCCTC | 1 |
| oadh-F6 | TGAGGTTCTGACTGCTCATCTCGCGTCTTCGGCCATCTCCTC | 1 |
| oadh-F7 | GAAGACGCGAGATGAGCAGTCAGAACCTCACCATCGTGCAGG | 1 |
| oadh-F8 | CTCCGAACTCGTCCCAGAGGCCCTCC | 1 |
| 1752-lrp (pKS13) P1 | TCACTATAGGGCGAATTGGGTACCGGACGGCGAAGACGGTTAG | 1 |
| 1752-lrp (pKS13) P2 | AGTCGAGAGCCGAGACAGAATTCATAACTCCCGAAAGGACAATC | 1 |
| 1752-lrp (pKS13) P3 | GGAGATGGCCGAAGACGCGAGATGAGATACGACCGCCTCGAAG | 1 |
| 1752-lrp (pKS13) P4 | GTGGCGGCCGCTCTAGAACTAGTGGGGTGGCGAAGTTGACCTC | 1 |
| pKL3 1752 P2 | CGAGGGGTTTTATCCACGGCATGCCAACTCCCGAAAGGACAATC | 1 |
| pKL3 for | GGCATGCCGTGGATAAAAC | 1 |
| Ana 10nt Del for neu | CGGCCCTCACCGACTAC | 2 |
| Ana 10nt Del rev neu | TCGACGTGGTCCTGCATC | 2 |
| A0326 Ana fdx for | GACAGTTAAGCCGTTCATGTG | 2 |
| Ana fdx rev | CGCGTCAGTTCGTAGTAGTC | 2 |
| trpA for | TACGTCGTGGTCAACGTTTC | 2 |
| trpA rev | GCCGACGTTGATATGGAAGG | 2 |
| 2524 P1 | CCGAACTGGACCGAACTG | 1, 2 |
| 2524 P2 | TCACAGGTCGAGGCGCGCCTCGACCTGTGATTC | 1 |
| 2524 P3 | GAAATCACGCCGTCATGACGGCGTGATTTCTCC | 1 |
| 2524 P4 | GGAGCGTCTTCGAGATGG | 1, 2 |
| 2528 21nt P1 | TCCATCAGGCGCTCCATGTC | 1 |
| 2528 21nt P2 | CTCGAACACGTCGGGCATGTCGAACCGGAAGCCG | 1 |
| 2528 21nt P3 | TTCCGGTTCGACATGCCCGACGTGTTCGAGCG | 1 |
| 2528 21nt P4 | GGCGAGGTCGACGATAGC | 1 |
| Ana 2528 for2 | CCATCAGGCGCTCCATGTCG | 2 |
| Ana 2528 rev (neu) | TTGACGCCGTAGAGAAAGAC | 2 |
| Ana 2528_21 for3 | GCGACGGCTTCCGGTTCGAC | 1, 2 |
| Ana 2528_21 rev2 | TCGGGCCGCTTGCCGAAGTAG | 2 |
| P1 (2528fw) mit HindIII | GACTAGAAGCTTTCTTTGACCGCCGCGAAGAC | 1 |
| P6 (2528rv) mit BamHI | GACTAGGGATCCAGTCGAGAAGCGGCGTCGTC | 1 |
| 2528 Mut. Stoppcodon Fw | CCAGCAGATAATGCAGTAAACGCTGGTGTTCCTCG | 1 |
| 2528 Mut. Stoppcodon Rv | CGAGGAACACCAGCGTTTACTGCATTATCTGCTGG | 1 |
| P1 (2528) | TCTTTGACCGCCGCGAAGAC | 1 |
| P2 (2528) | CGCGAGTTCGTCCATGTCCGTTCGTAAGGACGTAGAG | 1 |
| P3 (OE3381R) | TCCTTACGAACGGACATGGACGAACTCGCGGGGACATC | 1 |
| P4 (OE3381R) | CGGCCATATCGGCGCTCAGCGCGCGTCGGCGAG | 1 |
| P5 (OE3381R) | GCCGACGCGCGCTGAGCGCCGATATGGCCGCACAG | 1 |
| P6 (2528) | AGTCGAGAAGCGGCGTCGTC | 1 |
| P2 Sigma38 | TGTTCTACTAAGGCCAACTCGCGGCCGTCTTCGGTG | 1 |
| P3 Sigma38 | AGACGGCCGCGAGTTGGCCTTAGTAGAACAGGAACC | 1 |
| P4 Sigma38 | ACCTGGTCGCAGAGGTTGGCGTTCAGCTCGAACAG | 1 |
| P5 Sigma38 | CGAGCTGAACGCCAACCTCTGCGACCAGGTCGTCTC | 1 |
| P6 Sigma38 | GGAGCAGCGCCGTCTGGAAC | 1 |
| P2 (ara) | CCCAAACACAACCATGTCCGTTCGTAAGGACGTAGAG | 1 |
| P3 (ara) | TCCTTACGAACGGACATGGTTGTGTTTGGGAATGTTTCTG | 1 |
| P4 (ara) | CGGCCATATCGGCGCTCATGATGATGATACTGTTGCCTCC | 1 |
| P5 (ara) | GTATCATCATCATGAGCGCCGATATGGCCGCACAG | 1 |
| Ana 2528_21nt for | CGCTGTTGGAGAAGAACG | 2 |
| Ana Stopp 2528rev | CGGGCGAGTCGGTGTGGAAC | 2 |
| Ana Stopp 2528for | TGTCGCTCGTCGGCTCGATG | 2 |
| M13-20 | GTAAAACGACGGCCAGTG | 3 |
| M13 | GGAAACAGCTATGACCATG | 3 |

References

Allers T, Ngo H-P, Mevarech M, Lloyd RG (2004) Development of additional selectable markers for the halophilic archaeon Haloferax volcanii based on the leuB and trpA genes. *Appl Environ Microbiol* 70: 943–953. https://doi.org/10.1128/aem.70.2.943-953.2004

Ludt K (2018) *Polyploidie in Prokaryoten.* PhD thesis. Goethe-University, Frankfurt, Germany
